# Discordant phenotype caused by *CASK* mutation in siblings with *NF1*

**DOI:** 10.1038/s41439-019-0051-0

**Published:** 2019-04-26

**Authors:** Hiroaki Murakami, Yuichi Kimura, Yumi Enomoto, Yoshinori Tsurusaki, Moe Akahira-Azuma, Yukiko Kuroda, Megumi Tsuji, Tomohide Goto, Kenji Kurosawa

**Affiliations:** 1 Division of Medical Genetics, Kanagawa Children’s Medical Center, Yokohama, Japan; 2 Clinical Research Institute, Kanagawa Children’s Medical Center, Yokohama, Japan; 3 Division of Neurology, Kanagawa Children’s Medical Center, Yokohama, Japan

**Keywords:** Disease genetics, Disease genetics

## Abstract

With the advent of next-generation sequencing (NGS), a blended phenotype has been shown to be caused by multilocus molecular diagnosis. Here, we present siblings of neurofibromatosis type 1 (NF1) with discordant phenotypes. Further genetic investigation revealed that the younger sister had trisomy 8 mosaicism with a low ratio and a known pathogenic mutation in the *CASK* gene. This is the first report of a blended phenotype caused by NF1, CASK disorder, and trisomy 8 mosaicism.

NF1 is an autosomal dominant disorder characterized by multiple café-au-lait spots, growth of tumors along nerves, macrocephaly, learning difficulties and other symptoms. NF1 is also characterized by considerable inter- and intrafamilial variability in phenotypic expression^1^. However, its underlying mechanism is not well understood. Here, we present a good case showing the usefulness of next-generation sequencing (NGS) to uncover the cause of intrafamilial phenotypic discordance of NF1.

The proband (patient 1) was a 5-year-old boy (III-2 in Fig. 1a). He was the first child of nonconsanguineous parents. He was delivered at 36 weeks of gestation after an uneventful pregnancy. His birth weight was 2776 g (+1.1 SD). He developed multiple café-au-lait spots after birth. He had more than ten macules over 5 mm in diameter by the age of 2 years. He started controlling his head at 4 months, sitting without support at 7 months, and walking unsupported at 18 months. He spoke his first word with meaning at 36 months and two-word sentences at 48 months. He had attended a local education center for the follow-up of his developmental delay (IQ = 54) and autistic behaviors. He was referred to our hospital at the age of 5 years. His karyotype was 46,XY. Brain magnetic resonance imaging (MRI) was normal. Upon physical examination at 7 years and 10 months, his weight was 17.6 kg (−0.5 SD), height 105.9 cm (−0.9 SD), and occipital frontal circumference (OFC) 53.0 cm (+1.2 SD). He did not have specific facial features except for the relative macrocephaly (Fig. 1b).

**Fig. 1:**
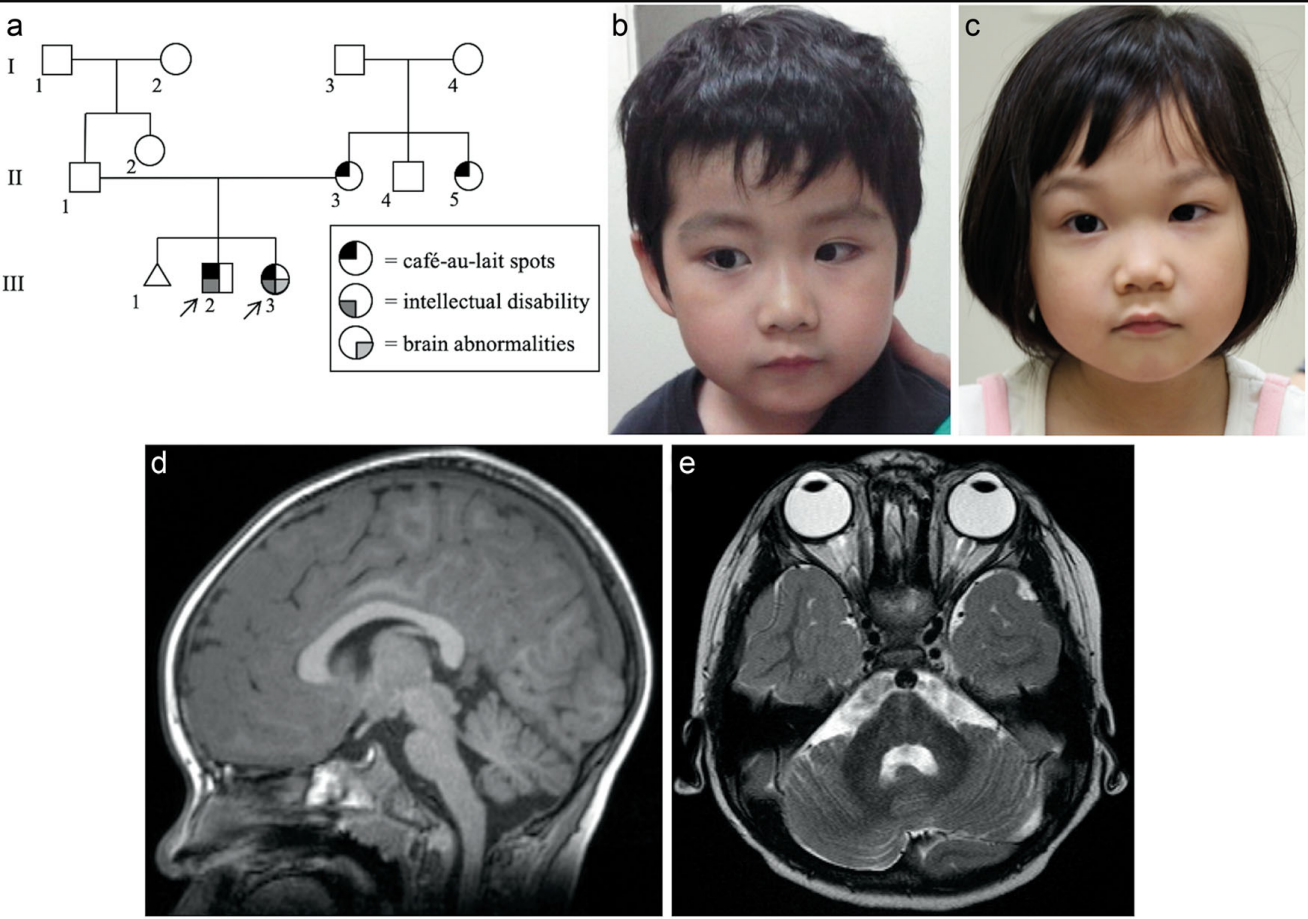
The family pedigree, photograph, and brain MRI of Patient 1 and 2. **a** Three-generation pedigree of the family in this study. Each symbol is annotated below. **b**, **c** Photograph of patients 1 and 2. Macrocephaly was noted in patient 1, but microcephaly and bulbous nose were noted in patient 2. **d**, **e** Brain MRI of patient 2 (**d** axial FLAIR image view, **e** sagittal T1-weighted view). MRI reveals mild hypoplasia of the cerebellum and brainstem. MRI magnetic resonance imaging

The younger sibling (patient 2) was a 5-year-old girl (III-3 in Fig. 1a). She was the second child of their parents and delivered at 40 weeks of gestation after an uneventful pregnancy. Her birth weight was 3068 g (−0.1 SD). She also had multiple café-au-lait spots after birth with seven macules over 5 mm in diameter. She began to control her head at 3 months, sit without support at 7 months, and walk alone at 16 months. She was referred to our hospital along with patient 1 at the age of 3 years for assessment of developmental delay. Upon examination, she could not speak any words with meaning. Her weight was 13.0 kg (−0.9 SD), height 94.3 cm (−0.8 SD), and OFC 46.0 cm (−1.7 SD). She was hypotonic. Her gait was ataxic with a widened base and truncal instability. Her behavior was autistic with restlessness, and she had difficulty making eye contact. She had a round face, sparse scalp hair, large ears, and downturned corners of her mouth (Fig. 1c). Her karyotype was mos 47,XX,+8[3]/46,XX[47]. Brain MRI revealed hypoplasia of the cerebellum and brainstem (Fig. 1d, e).

Written informed consent was obtained from the parents of the patients, in accordance with the Kanagawa Children’s Medical Center Review Board and Ethics Committee. Genomic DNA was extracted from peripheral blood samples of patients. Extracted DNA was captured using the TruSight One Sequencing Panel and sequenced on an MiSeq platform (Illumina, Inc., San Diego, CA, USA) with 151-bp paired-end reads. Exome data alignment, variant calling, and variant annotation were performed as previously described^2,3^. Copy number variations (CNVs) were assessed by analyzing the NGS data based on log-ratio analysis and the *z*-score of read depth (XHMM) on each exon.

We first examined the NGS data of patient 1 to elucidate any underlying genetic causes. The average coverage depth of the entire panel was 53.97 reads, and 96.1% of the targeted bases were covered with more than 10× sequence reads. We identified a novel frameshift mutation in *NF1* (NM_000267:c.6060dupA:p.Asn2021Lysfs*3). Sanger sequencing confirmed that patient 2 had the same mutation and that this mutation was inherited from their mother (Fig. 2a). To elucidate the cause of the discordant phenotype in the siblings, we further analyzed patient 2 using the TruSight One Sequencing Panel. The average coverage depth of the entire panel was 86.4 reads, and 99.4% of the targeted bases were covered with more than 10× sequence reads. We found a recurrent nonsense mutation in *CASK* (NM_003688:c.2041 C > T:p.Arg681*). Sanger sequencing confirmed that it was a de novo mutation. Patient 1 did not have this mutation (Fig. 2b). There was no apparent pathogenic CNV in either patient.

**Fig. 2:**
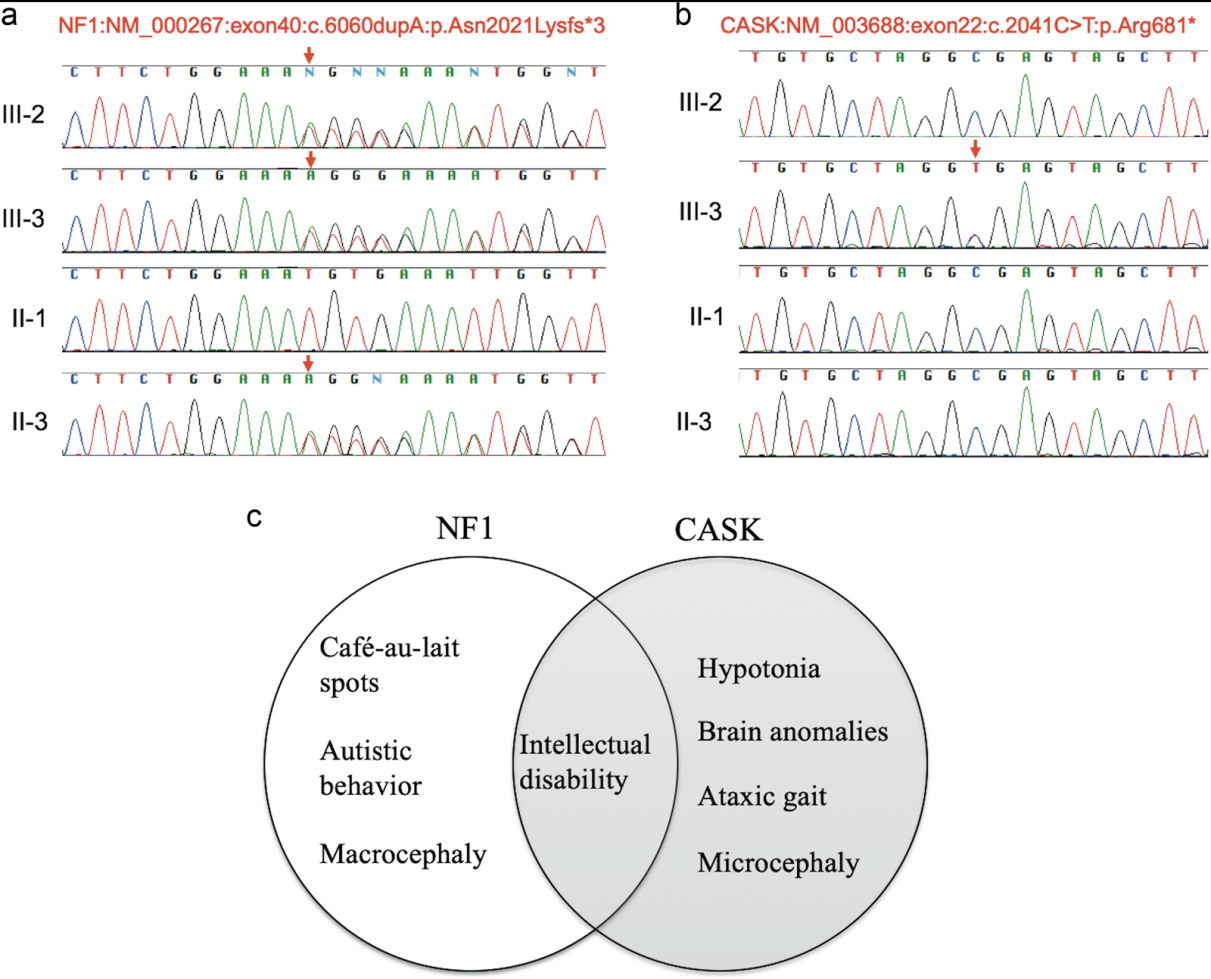
NF1 and CASK mutation. **a** *NF1* mutation. Electropherogram of the siblings and their parents. **b** *CASK* mutation. **c** Blended phenotype of patient 2 with *NF1* and *CASK* mutation

In this study, we demonstrated the discordant phenotypes among the siblings with café-au-lait spots and developmental delay. The younger sister had a more severe developmental delay and additional phenotypes, including ataxic gait, hypotonia, microcephaly, and hypoplasia of the cerebellum and brainstem. We identified a novel frameshift mutation in *NF1* of both siblings and their mother. The discordant phenotype of the siblings allowed us to consider the possibility of a blended phenotype caused by multilocus variations. We identified a de novo nonsense mutation in *CASK* of the younger sister. An X-linked dominant disorder of CASK is characterized by severe developmental delay, microcephaly, ataxic gait, and hypoplasia of the cerebellum and brainstem^4^. CASK belongs to the membrane-associated guanylate kinase (MAGUK) family and possesses a calcium/calmodulin-dependent serine protein kinase domain. It attaches to the membrane of synapses and functions in signaling pathways. CASK can also translocate to the nucleus and interact with transcription factors responsible for the expression of several genes required for normal brain formation^4,5^. In the context of phenotype−genotype correlation, we concluded that the *NF1* mutation underlay common phenotypes of the siblings, including café-au-lait spots and autistic behavior, but the *CASK* mutation contributed to the sister’s distinct phenotypes, including severe developmental delay, microcephaly, ataxic gait, hypotonia, and brain abnormalities (Fig. 2c). We could not exclude the possibility of somatic mosaicism for *CASK* mutation on the proband’s mother. However, we concluded that the *CASK* mutation of the sister was de novo because her mother did not possess any phenotypes caused by *CASK* mutations, including intellectual disability, ataxic gait, or microcephaly. Patient 2 had another possible pathogenic background, mosaic trisomy 8. However, she did not have typical phenotypes of mosaic trisomy 8, including scaphocephaly, frontal bossing, thin and long body trunk, and aberrant creases on the sole^6^. Although we concluded that mosaic trisomy 8 only slightly affected her phenotype considering the very low mosaic rate (6%), this may be the case for a variation in mosaic trisomy 8.

To our knowledge, this is the first case report of blended phenotypes caused by combinatory variants: frameshift mutation of *NF1* and nonsense mutation of *CASK*. The recent prevalence of NGS has unveiled the phenotypic variability of known genetic disorders and the presence of multilocus pathogenic variants in a single patient. Large-scale analyses of whole exome sequencing revealed that 3.5−4.9% of unselected cases have multilocus molecular diagnoses^7,8^. We should reevaluate the results of genetic analysis when we recognize atypical phenotypic expansion, especially in the case of disease characterized by inter- and intrafamilial phenotypic variability such as NF1.

## Data Availability

The relevant data from this Data Report are hosted at the Human Genome Variation Database at 10.6084/m9.figshare.hgv.2564; 10.6084/m9.figshare.hgv.2567.
